# Total delay and associated factors in pulmonary tuberculosis patients in Golestan province

**DOI:** 10.22088/cjim.11.1.67

**Published:** 2020

**Authors:** Nadia Alipour, Mahnaz Sheikhi, Jamshid Yazdani Charati, Hossein Mohsenipouya, Bizhan Shabankhani, Mohammad Sadegh Rezaii

**Affiliations:** 1 Student Research Committee, Faculty of Health, Mazandaran University of Medical Sciences, Sari, Iran; 2 Golestan University of Medical Sciences, Gorgan, Iran; 3 Department of Biostatistics, Faculty of Health, Health Sciences Research Center, Addiction Institute, Mazandaran University of Medical Sciences, Sari, Iran; 4 Faculty of nursing, Mazandaran University of Medical Sciences, Sari, Iran; 5 Research center for Hospital Infections, Mazandaran University of Medical Sciences, Sari, Iran; 6 Department of Biostatistics, Faculty of Health, Mazandaran University of Medical Sciences, Sari, Iran

**Keywords:** Tuberculosis, Logistic regression, Treatment, Delay

## Abstract

**Background:**

Delay in diagnosis and treatment of TB is a critical component in TB control program which thereby spreading illness in the community. Sicnce Golestan province has the high risk with high rates of tuberculosis in the country, therefore, the analysis of the factors associated with treatment delay in this province for effective interventions and proper planning is considered necessary.

**Methods:**

689 patients documents of TB cases in the health department of Golestan University of Medical Sciences in 2016 were enrolled in this survey. The response variable in this study was having the delay or not (via determining the 34 day as cut-off point in the interval between the date of onset of the symptoms and the date of treatment start-up). The data were analyzed using SPSS 24 software and final significant level for multivariate logistic regression model was considered 0.05.

**Results:**

Median (mean) treatment delay was calculated 49(77.75) days. In the current study 60.4% of patients had total delay greater than 34 days. In final model variables such as type of PTB (OR=0.645), contact history (patients who had no contact with TB patients (OR=1.441)) and patients who their contact history were unknown (OR=1.654)) had significant relationship with delay in starting treatment after 34 days of onset of symptoms of PTB patients in Golestan (p<0.05).

**Conclusion:**

It should beam emphasis on increasing the community's awareness of the symptoms of tuberculosis and effective collaboration should be made between the Infectious Disease Control Center and the private and public sectors.

Pulmonary TB still imposes a highburden on global health (1). The most important issue in TB control is to attempt for timely and accurate diagnosis and treatment of TB patients (2). Delay in diagnosis and treatment of tuberculosis is an important factor in TB control (3). An infected person can transmit the disease to 10-15 people on average per year, thereby, spread the disease in the society. Subsequently, delay can exacerbate the condition, cause complexity of the patient's condition leading to increase the risk of death (4-7). Based on the report of the Infectious Diseases Center at Ministry of Health, the incidence rate of TB in Iran is 12.59 per 100,000 people and among the provinces, Golestan and Sistan and Baluchistan with 41.6 and 38.26 per 100,000 people had the highest TB incidence rate, respectively. Also, the rate of case detection has been reported to be 60% in Iran (8), while the rest of patients will spread the infection in the community until they are detected and treated. Therefore, analysis of associated factors with unacceptable delay at the start of treatment after onset of the symptoms (more than 34 days total delay) in TB patients in Golestan province seems to be necessary for effective interventions and proper planning due to the high rate of TB incidence in this province.

## Methods

The present study is a Routine Data Analysis; data were extracted from TB patients’ documents that referred to health centers affiliated to the Golestan University of Medical Sciences and Health Services in 2016. The data of 793 TB patients were available in Golestan province in 2016, of which 689 patients had smear tests result which were included in the study. The recorded information included age, gender, nationality, place of residence, prison status, type of PTB, history of TB contacts, employment status, education level, result of HIV test and marital status. The response variable in this study was having total delay/not having total delay (with a 34 days cut-off point (9)) in PTB patients. The delay in this study was defined as the time from onset of the symptoms to initiation of the treatment (total delay) in PTB patients. For assessing the associated risk factors with response variable, we used logistic regression model. 

As other regression models, it is used to achieve an appropriate model for investigating the relationship between the response variable (categorical variable with two or more states) with one or a set of independent variables (9). To select variables for multivariate logistic regression model, first all of the variables were evaluated using univariate regression model and then variables with<0.3 significance level were entered into the model. For analyzing the data we used the effect size of the regression coefficients and confidence intervals and also for interpreting the risk factors on the delay in the onset of the treatment, we used odds ratios which were obtained from data analysis. Data were analyzed with SPSS 24 and significance level was considered 0.05.

## Results

There were 348 (50.5%) males among the total PTB patients. The mean age of patients was 20.46±50.14 years and was 51.26±19.86 and 49±21.03 years for males and females, respectively. Most of the patients (36.6%) belonged to the >60 years age category. In this study, 56.9% of the patients lived at the rural areas. Only 386 (56%) patients had performed HIV test, of which 9 and 377 patients’ test were positive and negative, respectively. A high percentage (63.1%) of patients had smear-positive PTB. The median (mean) of total delay, delay in treatment, delay in diagnosis was 49 (77.75), 0 (0.53) and 49 (77.81) days, respectively. Based on the data, 60.4% and 91.7% of the patients had >34 days and <6 months total delay, respectively. On the other hand 3.2%, 9.4%, 31.3% and 62.4% of patients had <one week, <2 weeks, < one month, and <60 days delay in diagnosis, respectively. Based on the calculated treatment delay, 99% of the patients had less than one week delay in treatment and 79.7% of the patients had no delay in diagnosis until beginning of the treatment. More descriptive information of PTB patients in Golestan province was shown in table 1.

According to the univariate logistic regression analysis, variables such as nationality (OR=0.55), place of residence (OR=0.649), history of contact with PTB patient (patients with no PTB contact history, OR=1,475), patients with unknown PTB contact history (OR=1.83)), HIV test status (OR=1.22), educational level (patients with high school education, OR=1.282), patients with academic education (OR=1.769), type of pulmonary PTB (OR=0.626), and the age group of patients were significantly correlated with delay in the onset of PTB treatment. This means that delay in onset of the PTB treatment is 55% lower in non-Iranian patients than Iranian patients. Also, patients which were residents in rural areas are 65 percent less likely to have>34 days total delay than those who reside in cities. Patients with no history of contact with PTB patients and those with unknown TB contact history were 47% and 83% more likely to have delay in treatment exceeding 34 days than those with a TB contact history, respectively. 

The education variable also had a significant relationship with unacceptable total delay, so that with increasing educational level of the patients, the odds of delay in onset of the treatment after 34 days were increased, so patients with high school education and academic education had 28% and 77% more chance to have >34 days total delay with respect to the illiterate patients, respectively. There were no significant relationships between gender, marital status, prisoner status, and job categories of the patients with the response variable (table 2).

In this step, significant variables in the univariate model and those with<0.3 p-value were included into the multivariate logistic regression model. Using the univariate logistic regression model, variables such as nationality, educational level, type of PTB, region of residence, HIV test status, history of contacts with PTB patients, and age groups were introduced into the multivariate logistic regression model. 

The variables such as nationality and educational level of the patients were not in the final model and variables such as HIV test status and age of patients were not significant in the multivariate analysis. Based on the results of multivariate logistic regression model, variables such as type pulmonary PTB, place of residence, and history of contacts with TB patients had significant relationships with more than 34 days total delay. The details of significance, odds ratios and confidence intervals of the variables in multivariate logistic regression model were shown in table 3.

**Table1:** Demographic characteristics of PTB patients in Golestan province in 2016

**Characteristic**		**N (%)**	**Characteristic**		**N (%)**
Age	<5	1(0.1%)	Gender	Male	348(50.5%)
	6-14	16(2.3%)		Female	341(49.5%)
	15-24	60(8.7%)	Prisoner status	Prisoner	26(3.8%)
	25-39	170(24.7%)		Non-prisoner	663(96.2%)
	40-60	190(27.6%)	Nationality	Iranian	663(96.2%)
	>60	252(36.6%)		Non-Iranian	26(3.8%)
Marital status	Single	116(16.8%)	Type of PPTB	Smear Positive	435(63.1%)
	Divorced	18(2.6%)		Smear Negative	254(36.9%)
	Widow	96(13.9%)	Place of residence	urban	296(43%)
	Married	459(66.6%)		Rural	392(56.9%)
History of contacts with TB patient	Yes	224(32.5%)	HIV test result	Positive	9(1%)
	No	56(8.1%)		Negative	377(55%)
	Unknown	408(59.2%)		Unknown	303(44%)
Educational level	Illiterate	320(46.6%)	Job classification	Armed forces	3(0.4%)
	Elementary	165(23.9%)		Office workers	7(1%)
	Middle School	115(16.7%)		Service and sales staff	9(1.3%)
	High school	59(8.6%)		Farmers and fishermen	35(5.1%)
	Academic	30(4.4%)		Craftsmen	1(0.1%)
Type of patients	New case	618(89.7%)		Operating staff, assemblers of machinery, drivers	2(0.3%)
	Relapsed	43(6.2%)			
	Others	28(4.1%)		Worker	120(17.4%)
Clinical signs	fever	505(73.29%)		Other	512(74.3%)
	Chronic cough	476(69.08%)	Treatment outcomes	Error in diagnosis	21(3.0%)
	weight loss	349(50.65%)		Cured	366(53.1%)
	Blood in the sputum	173(25.11%)		In Treatment period	18(2.6%)
	Weakness or fatigue	395(57.32%)		Died	33(4.8%)
	Reduce/No appetite	385(55.88%)		Treatment failure	22(3.2%)
	Sweating at night	420(60.96%)		Treatment completed	221(32.1%)
	dyspnea	373(54.14%)		Others	8(1.2%)

**Table 2:** Evaluation of factors associated with total delay occurrence in pulmonary tuberculosis patients in Golestan province using univariate logistic regression

**Variable**		**B**	**SE**	**Z**	**Sig.**	**Exp(B)**	**95% CI for Exp(B)**	
Gender	Female=1/ Male=0	0.051	0.156	0.108	0.742	1.053	0.776	1.429
Nationality	non-Iranian=1/ Iranian=0	-0.599	0.401	2.225	0.136	0.550	0.250	1.207
Prisoner status	prisoner=1/Non-prisoner=0	0.223	0.420	0.282	0.595	1.250	0.549	2.846
Job category	self-employed=1/ employee=0	-0.432	0.695	0.387	0.534	0.649	0.166	2.532
Marital status	Single (Ref. cat)							
	Divorced	0.209	0.518	0.163	0.686	1.233	0.446	3.406
	Widow	0.009	0.278	0.001	0.975	1.009	0.585	1.740
	Married	0.260	0.210	1.529	.216	1.297	0.859	1.959
Education level	Illiterate (Ref. cat)							
	High school	0.248	0.159	2.429	0.119	1.282	0.938	1.751
	Academic	0.571	0.414	1.898	0.168	1.769	0.786	3.983
Type of PTB	smear positive=1/ smear negative=0	-0.469	0.165	8.060	0.005	0.626	0.453	0.865
Place of residence	rural=1/urban=0	-0.433	0.159	7.360	0.007	0.649	0.475	0.887
HIV test status	do not done=1/ done=0	0.199	0.158	1.597	0.206	1.220	0.896	1.662
History of contacts with TB patient	Had (Ref. cat)							
	Unknown	0.604	0.316	3.656	0.056	1.830	0.985	3.399
	Had not	0.389	0.169	5.308	0.021	1.475	1.060	2.053
Age group	< 25 (Ref. cat)							
	25-39	0.404	0.277	2.132	0.144	1.498	0.871	2.577
	40-60	0.675	0.275	6.020	0.014	1.964	1.145	3.368
	>60	0.310	0.261	1.412	0.235	1.364	0.817	2.277
Treatment outcomes	Treatment failure (Died + Treatment failure) (Ref. cat)							
	Cured (cured + Treatment completed)	-0.170	0.293	0.336	0.562	0.844	0.475	1.498
	Others	0.102	0.416	0.060	0.807	1.107	0.489	2.502
Type of patients	New case(Ref. cat)							
	Relapsed	-0.434	0.387	1.258	0.262	0.648	0.303	1.383
	Others	0.089	0.326	0.075	0.784	1.094	0.576	2.070

**Table 3:** Evaluation of factors associated with total delay occurrence in pulmonary tuberculosis patients in Golestan province using multivariate logistic regression

**Variable**		**B**	**SE**	**Z**	**Sig.**	**EXP(B)**	**95% CI for EXP (B)**	
Type of PTB	smear positive=1/ smear negative=0	-0.439	0.169	6.762	0.009	0.645	0.463	0.898
Place of residence	rural=1/urban=0	-0.409	0.162	6.349	0.012	0.664	0.483	0.913
HIV test status	do not done=1/ done=0	0.199	0.158	1.597	0.206	1.220	0.896	1.662
History of contacts with TB patient	Had(Ref. class)							
	Unknown	0.503	0.322	2.441	0.118	1.654	0.880	3.111
	Had not	0.365	0.172	4.514	.034	1.441	1.029	2.019
Age group	< 25 (Ref. cat)							
	25-39	0.363	0.281	1.666	0.197	1.438	0.828	2.495
	40-60	0.678	0.280	5.861	0.015	1.969	1.138	3.408
	> 60	0.322	0.267	1.451	0.228	1.379	0.817	2.328

## Discussion

There are several risk factors for delay in onset of the treatment in PTB patients. In the current study, we assess the relationship between risk factors and more than 34 days delay in onset of the symptoms until starting treatment. According to the results, type of pulmonary TB (smear positive, smear negative), place of residence, and history of contacts with TB patients had significant relationships with total delay (p<0.05). In addition to univariate analysis, age, nationality, educational level and HIV test status were considered as risk factors, but in multivariate analysis there were no significant relationships between them.

In this study, mean (median) interval for the onset of the symptoms until start of the treatments was estimated 109.58±77.75 (49) days, which is more than delays reported in Kurdistan province with 53 days (10), Mazandaran province with 69.9 days in average and 36 days median total delay (11), Argentina with 58 days (12), Qatar with 30 days (13), in Bangalore in India with 41 days median total delay (14), and Angola with 45 days median delay(15). By contrast, the obtained total delay in Golestan province was less than some areas, Markazi province with 129.9±171 days in average (16), Mashhad with 44.7 ± 99 days(17), Iran with 127±114 days in average and 44 days median delay (18), Ethiopia with 70.5 median total delay (19), New York with 57 days median total delay (20), France with 68 days (21), Norway with 63 days (22), Turkey with 49 days (23), Ghana with 104 days (24), Tanzania with126 days (25), India with 81 days median delay (26), West Kenya with 44 days median delay (27), Botswana with 12 weeks median delay and 17.3 weeks in average(28).Differences in delays in different studies may be because of various under study populations. The patients in the present study included patients whose records were collected in health centers whereas under study populations in other countries concluded the patients who referred to the hospitals it could be the reason for the differences in studies.

In the present study, the median total delay in women (50 days) was greater than men (47.5 days) which was agreeable with such studies (30-34). This result can be due to the women's dependence on their husbands, financially and follow-up treatment and transfer to treatment centers (35) but based on the multiple logistic regression, there were not significant relationships between gender and total delays and in contrast, univariate logistic regression showed 5% more odds to have delay in women than men. Also in a study which conducted in Mashhad, there were not significant relationships between gender and total delays (17). 

According to our results, 32.5% of patients had history of contact with TB patient and in 59.2% of cases that was unknown. On the other hand, having history of contacts with TB patients had significant relationship with unacceptable total delay. Odds ratio were 1.654 and 1.44 for patients with unknownTB contact history and those without a TB contact history, respectively, meaning that those categories have 65% and 44% more chance to have over 34 days total delay with respect to the patients who had history of contact with TB patients. Therefore, having a history of contact with TB patient was a reducer factor for delay in the beginning of treatment interval, that it can be due to increasing awareness and information of the patients with the disease symptoms leading to faster referral and quicker onset of the treatment in such patients.

In the current study, patients with over 60 years old category had the highest percentage of the patients and patients in 40-60 years age category had the highest total delay (57.5 day median delay), while there were no significant relationships between age groups and total delays. In the univariate analysis, all age groups had above 34 days total delay compared to the under 25 years old. The highest odds ratio belonged to 40-60 years age group (OR=1.964), this result may be due to this fact that this age group has more contact with people than those under 25 years old patients, or it can be indicative of a strong relationship between TB and HIV and AIDS. On a study conducted in Ethiopia patients with 15-34 years of age had the highest delay (36). A study in Argentina showed that those with over 50 years old were associated with total delays (12) and similar to our result, there was no relationship between age groups and total delays in Mashhad (17).

In the present study, a high percentage of patients (56.9%) lived in villages and there was a significant relationship between place of residence and total delay (OR=0.664), meaning that rural patients were 66% less likely to have more than 34 days total delay on the other hand, a high percentage of patients lived in rural areas and it can indicate their accessibility to health facilities and better economic conditions than those living in cities, this result was agree able with the result of Yazdani et al. (11, 37) and our result is in contrast with a study done in Mashhad and in their study, there was no significant relationship between urban residency and total delay (17).

Based on our analysis, there was no significant relationship between marital status of patients and delay in the onset of treatment. But based on the descriptive analysis, married patients had 1.3 times more probability to have more than 34 days total delay than single patients. Likewise, a study conducted in Kurdistan showed that delay in the beginning of treatment in single patients was less than the married patients (10), which it can be due to the dependence of married patients on their spouses. In the present study, 56% of patients had done HIV test, of which 1% was HIV positive. In a study conducted which in Mazandaran province in 2010, 89.2% of patients had not done HIV test (37). In another study in Mazandaran province during the 2009-2015, 82.23% of patients had not performed HIV test (11), decreasing this percentage in this study indicating increase in patients'knowledge about the disease and tendency to perform HIV test. Based on the analysis, there was no significant relationship between having more than 34 days total delay and HIV test status, but based on the univariate logistic regression analysis(OR=1.22) patients who did not perform HIV test had 22% more likely to have over 34 days delay than those who did HIV test.

In conclusion according to the results of this study, a high percentage of PTB (60.4%) patients had an unacceptable total delay (> 34 days).With respect to the high incidence rate of the PTB in Golestan province and the importance of the early diagnosis and initiation of treatment at the onset of disease, increasing the awareness of the people in the community has the high importance. Lack of attention to the timely treatment of these patients increase burden of diseases and its transmission in the community and imposes more costs on society and health system.According to the conducted studies on delay inTB diagnosis and treatment in Iran, the healthcare system has the greatest contribution in delay in treatment (18), therefore, there should be an effective and important collaboration between the center of contagious diseases and private and public sectors.Also the physicians' knowledge and skills about examination and suspicion onTB symptoms should increase and training and retraining courses for physicians and TB health care providers should be considered at the regular intervals.
